# Knowledge, attitudes, and practices related to COVID‐19 infection, related behavior, antibiotics usage, and resistance among Syrian population: A cross‐sectional study

**DOI:** 10.1002/hsr2.833

**Published:** 2022-10-03

**Authors:** Sarya Swed, Sheikh Shoib, Mohammad B. Almoshantaf, Walaa Hasan, Yomna E. Dean, Yousef Tanas, Haidara Bohsas, Hidar Alibrahim, Mohammad M. Hasan, Weaam Ezzdean, Hazem S. Ghaith, Lina T. Khairy, Agyad Bakkour, Ali Hadi Hussein Muwaili, Fatima A. A. Abdelmajid, Mhd K. Albuni, Elias Battikh, Dhuha Hadi Hussein Muwaili, Rima Qattea, Karam R. Motawea, Bisher Sawaf, Nashaat Kamal Hamdy Elkalagi, Safaa M. A. Ahmed, Hani Aiash, & Data Collection Group

**Affiliations:** ^1^ Faculty of Medicine Aleppo University Aleppo Syria; ^2^ Department of Psychiatry Jawahar Lal Nerhu Memorial Hospital (JLNMH) Srinagar India; ^3^ Department of Neurosurgery Ibn Al‐Nafess Hospital Damascus Syria; ^4^ Clinical Oncology and Nuclear Medicine Suez Canal Egypt; ^5^ Faculty of Medicine Alexandria University Alexandria Egypt; ^6^ Department of Biochemistry and Molecular Biology, Faculty of Life Science Mawlana Bhashani Science and Technology University Tangail Bangladesh; ^7^ Department of Urology Ibn Al‐Nafees Hospital Damascus Syria; ^8^ Faculty of Medicine Al‐Azhar University Cairo Egypt; ^9^ General Practitioner The National Ribat University Al‐Ribat Sudan; ^10^ Faculty of Medicine Albaath University Homs Syria; ^11^ General Practitioner Ivano‐Frankovsk National Medical University Ivano‐Frankivsk Oblast Ukraine; ^12^ General Practitioner University of Medical Sciences and Technology Khartoum Sudan; ^13^ Department of Internal Medicine Damascus University Damascus Syria; ^14^ Department of Internal Medicine Syrian Private University Damascus Syria; ^15^ Internal Medicine and Tropical Medicine at Faculty of Medicine Al Arish University Al Arish Egypt; ^16^ Faculty of Medicine Shendi University Shendi Sudan; ^17^ Cardiovascular Perfusion, Medicine, Surgery Upstate Medical University Syracuse New York USA; ^18^ Family Medicine Suez Canal university Ismailia Egypt

**Keywords:** antibiotics assistance, COVID19, KAP, Syria

## Abstract

**Background and Aims:**

Antibiotic resistance is seen as a worldwide health risk as a result of the overuse of antibiotics. Many countries noted that antibiotic usage was high during the COVID‐19 pandemic. The purpose of this study is to evaluate Syrians' knowledge, attitudes, and practice about the use of antibiotics and antibiotic resistance during the COVID‐19 epidemic.

**Methods:**

A cross‐sectional study was conducted using an online questionnaire to collect the data from the Syrian population from February 5 to March 4, 2022. Syrians 18 years or older all over the world were able to participate in this study. A convenience snowball sampling method was used. SPSS version 20.0 was used to analyze the data. To examine the results, binominal logistic regression was used. Statistical significance was defined as a *p* < 0.05.

**Results:**

Out of 2406 respondents, 60.2% knew that transmission of COVID‐19 could occur even if the patient has not developed any symptoms, and 91.6% were able to recognize the main clinical symptoms of COVID‐19. There was a statistically significant difference between male and female knowledge of COVID‐19 (*p* = 0.002), with males having 3.78 ± 2.1 (2.7–3.87) and females scoring 3.93 ± 2.3 (3.7–4.1). Newly graduated students have more knowledge of COVID‐19 than other subtypes of Job (*p* = 0.0001), and those with medical practice are more knowledgeable than those without (*p* = 0.0001). Only 16.6% answered that taking antibiotics would not speed up the recovery from all the infections. 65.3% answered correctly that misuse of antibiotics could cause antibiotic resistance.

**Conclusion:**

Our study concluded that the Syrian population demonstrated good knowledge of COVID‐19 and moderate acceptance of the new norm. Knowledge regarding antibiotic use and resistance and practice of preventive measures was poor, which can encourage the health authorities to develop community education programs to increase public awareness of the usage of antibiotics and safety protocols during the COVID‐19 pandemic.

## 1 INTRODUCTION

Coronavirus disease is a pathogenic viral infection brought on by the highly contagious SARS‐CoV‐2 virus, which was first discovered in Wuhan, the city of Hubei, China, in 2019.^1^, ^2^ SARS‐CoV‐2 is one of the coronaviruses; like other human coronaviruses, it has a single‐stranded, positive‐sense RNA genome and infects people by binding to the ACE2 receptor on the surface of their cells.^3^, ^4^ The COVID‐19 virus is spread through infected people's droplets in the air.^5^ The WHO received reports of a total of 6.09 million fatalities and 472.8 million confirmed cases up through March 2022.^6^ In Syria, the number of confirmed cases and deaths were 55,595 and 3000, respectively.^6^, ^7^ The clinical signs and symptoms of COVID‐19 might vary from an asymptomatic infection to a serious sickness needing hospitalization and oxygen support.^8^ Patients with mild to moderate COVID‐19 might experience fever, cough, sore throat, diarrhea, fatigue, fatigue, headache, muscle or joint pain, and loss of smell and taste.^9^ At first, the treatment was limited to symptomatic and supportive measures. In 2021, therapeutic medications, including antiviral (e.g., remdesivir, Paxolvid and Molnupiravir) and supporting agents (corticosteroids, IL‐6 antagonists), became available.^10^, ^11^ Anti‐SARS‐CoV‐2 monoclonal antibodies (Bamlanivimab plus etesevimab, casirivimab plus imdevimab, and sotrovimab) have been authorized for the treatment of mild to moderate COVID‐19 cases that have not yet required hospitalization but are at a high risk of developing into severe illness and/or inpatient care.^12^ Antibiotics have been crucial in treating and controlling infectious illnesses since their discovery and have helped save countless lives.^13^ However, in general practice, antibiotic misuse has led to difficulty in treating common infections, due to antibiotic‐resistant bacteria that take longer to resolve and increase the burden on health care systems.^14^, ^15^ It is anticipated that this issue will worsen in developing nations where infectious illness is common, there is little access to healthcare, and regulations are weak.^13^ In a cross‐sectional study in Syria, 87% of pharmacies agreed to sell antibiotics without prescription.^16^ Another study in Syria revealed that 85% of people used antibiotics within 4 weeks; only 43% of them were prescribed the antibiotic by a physician, while 57% used an old prescription or nonmedical advice to get the antibiotic.^17^ This phenomenon can be attributed to poverty, low socioeconomic status, lack of health awareness, and limited health resources, especially after the war.^18^, ^19^ According to a Malaysian study, in the early stages of the COVID‐19 epidemic, antibiotic use was not very common; only 17.1% of people used antibiotics, with 5.5% of patients receiving two or more antibiotic kinds^20^ In 2021, 78% of COVID patients used systemic antibiotics other than macrolides; 72% used beta‐lactams, 13% used quinolones, and 2.2% used linezolid.^21^, ^22^ During the COVID‐19 pandemic in Syria, there was a significant increase in using antibiotics as well.^19^ The knowledge, attitudes, and practices (KAP) of the public regarding the COVID‐19 pandemic are essential. Therefore, researchers from Malaysia and Ethiopia investigated and found that the early phase of the pandemic was largely favorable.^23^, ^24^ KAP surveys may be used to find out additional information that will aid in the development of public education materials and to discover knowledge gaps, behavioral trends, or cultural attitudes.^23^ A Malaysian Survey of Knowledge and Awareness reported that only 36.8% of respondents knew that taking antibiotics has no role in speeding up the recovery process of all infections.^23^ Another research in Southeast Ethiopia found that 50.6% of the participants had adequate knowledge of the general drug consumption in COVID‐19.^1^ The purpose of this study is to assess Syrian community KAP about antibiotic usage and resistance during the COVID‐19 pandemic.

## 2 METHODS

### 2.1 Study design

A web‐based survey was utilized to gather information from Syria for an observational cross‐sectional research. The survey was created using data from a prior Malaysian research,^23^ after which it was revised and translated to reflect the Syrian situation. The survey was sent out to 30 individuals for completion to prevent errors and ensure that it was understandable to all participants. After that, a pilot test with 50 participants was conducted to confirm the validity and reliability of the survey. The tool maintained high internal consistency, as shown by Cronbach's alpha values for the regions ranging from 0.712 to 0.861. (Cronbach's alpha was 0.766, 0.7122, 0.73, and 0.861 for the knowledge toward COVID19 scale, the scale of the preventive measure, the knowledge of antibiotics uses and resistance scale, and attitude toward new norms during the COVID‐19 pandemic scale, respectively). On the Google form website, 2467 Syrians were asked to take part in this survey, where the data collection was started from February 5, 2022, to March 4, 2022. This cross‐sectional study only included Syrians over 18 and did not include anybody under 18 or someone living outside Syria. We gathered the data we needed from the respondents using the convenience and snowball approaches. Several social media platforms, including Facebook, WhatsApp, Twitter, and Telegram, were used by the data gathering respondents, to publish the questionnaire to obtain a large sample. The sample size was estimated using Calculator.net, available at “https://www.calculator.net/sample-size-calculator.html.” The United Nations estimates that there will be roughly 18 million people living in Syria in 2019.^25^ Then, using a 0.05 margin of error and a 95% confidence level, we ran a statistical power analysis to get the sample size, and the minimum sample size appeared to be 385. The used questionnaire was uploaded as Supporting Information.

### 2.2 Measures

The 42 questions were separated into five parts on the questionnaire. The first question was about the acceptance for participation and completing the survey; thus, we removed the people who refused to fill the questionnaire.

### 2.3 Sociodemographic characteristics

This section includes nine questions about age [three age groups (18–29, 30–49 and >50) years], gender, the governorate of origin, income, job situation, level of education, and involvement in or interest in a healthcare‐related sector, as well as suffering from chronic disease.

Furthermore, we have four items scales in our study:

1. Knowledge of COVID‐19 pandemic
  The responses ranged from Correct, Incorrect, to Unsure (7 items).
2. Preventive measures during the COVID‐19 pandemic
  The replies were classified as “true” or “false” (10 items).
3. Knowledge of antibiotics use and resistance
  The responses ranged from strongly Correct, Incorrect to Unsure (10 items).
4. Attitude toward new norms during the COVID‐19 pandemic

There were a variety of replies, including strongly disagree, disagree, neutral, agree, and highly agree (7 items). The answers were re‐categorized into “correct,” “incorrect,” and “unsure,” including both domains of knowledge. Every correct response was given one point, while incorrect or unsure answers got zero. In the practice domain, each “yes” response was scored one point. Every strongly agreed or agreed response was given one point in the attitude domain. The following have been the minimum and maximum score ranges for each domain: COVID‐19 (0–7), antibiotics (0–10), practice (0–10), and attitude (0–7). A pooled score of above 80% for each category reflects strong knowledge, adequate practice, and a good attitude.

### 2.4 Statistical analysis

The SPSS version 20.0; IBM was employed to analyze the data and statistically significant considered at (*p*‐value < 0.05). All of the variables were analyzed in a descriptive form. The categorical results were reported as frequency and percentages, whereas means and standard deviations were used to report the continuous variables. One‐way analysis of variance (ANOVA) was conducted to determine if the KAP scores were different for sociodemographic characteristics. Data is presented as Mean± Standard Deviation (95% confidence interval: lower band‐upper band). To determine the influence of baseline factors on the chance that Syrian participants had considerable knowledge about the COVID‐19 pandemic and antibiotic usage and resistance, binominal logistic regression was used. To evaluate the association between KAP scores, a Pearson's item correlation was performed.

### 2.5 Ethics

The Aleppo University and the Damascus Medical Research Ethics Committee provided their clearance. Participants were given a special URL to access the online survey on Google form. Participants were asked in the first page of the survey if they were able to complete the survey and were referred to the participant information page, which contained information about the study, before answering the survey, so the participation was optional, and the replies were kept private. The volunteers were transferred to the online questionnaire after clicking “accept to participate.” Each participant may take about 12 min to complete the questionnaire. All of the replies were stored in a secure online database.

## 3 RESULTS

Two thousand four hundred and sixty‐seven participants were invited to solve the online questionnaire on the google form. Out of which 18 persons refused to participate in the survey, and 43 were under 18. Thus, only 2406 were applicable for statistical analysis; 45.3% of the answers were received personally, and 54.7% were received through social media.

Most of the respondents' (71.9%) ages were between 18 and 29 years old, whereas only 6.9% were above 50. The majority of the respondents were females (67.2%), and 51.4% of them have finished or reached their university stage or above such as a master's or PhD 46% of total respondents were students, and 44.4% have a medical education background. Nonetheless, only 10.5% have confirmed being diagnosed earlier with chronic disease. Characteristics of respondents are described in Table 1.

**Table 1:** Demographic characteristics (*n* = 2406)

Demographic variables	Frequency	Percentage
Age		
18–29	1729	71.9
30–49	510	21.2
Above 50	167	6.9
Sex		
Male	790	32.8
Female	1616	67.2
Education		
Primary or below	209	8.7
Secondary	961	39.9
Tertiary	1236	51.4
The job		
Full‐time (government)	353	14.7
Full‐time (private)	199	8.3
Student	1107	46
Unemployed	536	22.3
Retiree	50	2.1
New graduated	161	6.7
Medical education background		
Yes	1069	44.4
No	1337	55.6
Household income		
Bad (Under 50.000 SP*)	263	10.9
Moderate (50.000–100.000 SP)	955	39.7
Good (100.000–300.000 SP)	1045	43.4
High (Above 300.000 SP)	143	5.9
Chronic disease		
Yes	252	10.5
No	2154	89.5

*p < 0.05.

### 3.1 Knowledge of COVID‐19

The understanding of COVID‐19 among the respondents was evaluated using seven questions. The average score for knowledge was 5.22 (SD = 1.414, range 0–7). The total percentage of accurate responses was 74.5%. Most of the respondents could answer five out of seven questions correctly. However, only 60.2% knew that transmission of COVID‐19 could occur even if the patient has not developed any symptoms, and 91.6% were able to recognize the main signs of COVID‐19, but surprisingly 12.6% didn't realize that the COVID‐19 pandemic is of viral origin as 1.5% answered “incorrect,” and 10.1% were “not sure” about their answer (Table 2). Scores on the COVID‐19 knowledge test varied by gender, age group, and educational level, the job, medical education, household income and chronic diseases using one way‐ANOVA factor (Table 4). A statistically significant difference in COVID‐19 knowledge was indicated between males 3.78 ± 2.1 (2.7–3.87) and females 3.93 ± 2.3 (3.7–4.1) (*p* = 0.002). Newly graduated students had a greater understanding of COVID‐19 than other subtypes of the job (*p* = 0.0001), and those with medical practice had greater knowledge than those without medical practice (*p* = 0.0001). In addition, compared to those in other subcategories of household income, those with low household incomes had the lowest knowledge of COVID‐19 (Table 3).

**Table 2:** Descriptive data of knowledge toward COVID‐19

Item	Correct	Incorrect	Unsure
1. A virus is the origin of the COVID‐19 pandemic	2125 (88.3%)	37 (1.5%)	244 (10.1%)
2. Fever, cough, sore throat, and breathing difficulties are the predominant clinical signs of COVID‐19	2203 (91.6%)	62 (2.6%)	141 (5.9%)
3. COVID‐19 is highly contagious	2075 (86.2%)	107 (4.4%)	224 (9.3%)
4. Infected older adults, youngsters, those with comorbid conditions, and those with weak immune systems have higher difficulties	2003 (83.3%)	133 (5.5%)	270 (11.2%)
5. The COVID‐19 virus is mostly transmitted via respiratory secretions	1704 (70.8%)	664 (23.4%)	138 (5.7%)
6. Only once a person has symptoms may the COVID‐19 virus be transmitted	487 (20.2%)	1449 (60.2%)	470 (19.6%)
7. Over time, the COVID‐19 viral strain may change	1935 (80.4%)	61 (2.5%)	410 (17%)

**Table 3:** Differences in knowledge of COVID‐19, knowledge of antibiotics resistance, practice, and attitude score with demographic characteristics (one way‐analysis of variance)

Statement	Knowledge of antibiotics resistance		Knowledge of COVID‐19		Practise in preventive measures scores		Attitude scores		
	Mean ± SD (95% CI: Lower–Upper)	*p*‐value	Mean ± SD (95% CI: Lower–Upper)	*p*‐value	Mean ± SD (95% CI: Lower–Upper)	*p*‐value	Mean ± SD (95% CI: Lower–Upper)		*p*‐value
Age, years (Total) 18–29 30–49 Above 50	3.77 ± 2.19 (3.69–3.86) 3.82 ± 2.06 (3.7–3.9) 3.61 ± 2.51 (3.4–3.8) 3.84 ± 2.50 (3.4–4.2)	0.157	5.22 ± 1.41 (5.16–5.27) 5.33 ± 1.26 (5.27–5.39) 4.93 ± 1.64 (4.79–5.07) 4.68 ± 1.86 (4.57–5.14)	<0.0001*	4.8 ± 2.1 (4.7–4.9) 4.8 ± 2.0 (4.7–4.9) 4.9 ± 2.05 (4.7–5.1) 3.6 ± 2.7 (3.2–4.0)	<0.0001*	4.9 ± 1.6 (4.8–4.9) 4.8 ± 1.6 (4.7–4.9) 5.05 ± 1.7 (4.9–5.2) 4.8 ± 1.9 (4.5–5.1)		0.068
Gender (Total) Male Female	3.78 ± 2.1 (2.7–3.87) 3.93 ± 2.3 (3.7–4.1) 3.7 ± 2.2 (3.6–3.8)	0.023*	5.22 ± 1.41 (5.16–5.27) 5.25 ± 1.40 (5.15–5.34) 5.20 ± 1.42 (5.13–5.27)	0.442	4.8 ± 2.1 (4.7–4.9) 4.8 ± 2.1 (4.7–5.03) 4.7 ± 2.1 (4.6–4.8)	0.286	4.9 ± 1.6 (4.8–4.9) 4.7 ± 1.7 (4.6–4.8) 4.9 ± 1.6 (4.8–5.05)		0.002
Education (Total) Primary or below Secondary Tertiary	3.7 ± 2.2 (3.7–3.87) 4.1 ± 2.7 (3.7–4.4) 3.8 ± 2.1 (3.7–4) 3.7 ± 2.1 (3.6–3.8)	0.057	5.22 ± 1.41 (4.16–4.27) 4.57 ± 1.99 (5.30–5.85) 5.32 ± 1.30 (5.24–5.41) 5.24 ± 1.35 (5.16–5.31)	<0.0001*	4.8 ± 2.1 (4.7–4.9) 4.7 ± 2.3 (4.4–5.0) 4.8 ± 2.0 (4.7–4.9) 4.8 ± 2.1 (4.6–4.9)	0.882	4.9 ± 1.6 (4.8–4.9) 5.3 ± 1.8 (5.0–5.5) 4.8 ± 1.6 (4.7–4.9) 4.8 ± 1.6 (4.7–4.9)		0.001
The job (Total) Full‐time (government) Partial time (private) Student Unemployed Retiree new graduated	3.8 ± 2.2 (3.7–3.8) 3.4 ± 2.47 (3.2–3.7) 3.6 ± 2.4 (3.3–4) 4 ± 1.89 (3.8–4) 3.6 ± 2.4 (3.4–3.8) 4.1 ± 2.5 (3.4–4.8) 4 ± 2 (3.7–4.3)	0.002*	5.22 ± 1.41 (5.16–5.27) 5.15 ± 1.49 (4.99–5.31) 4.98 ± 1.58 (4.76–5.20) 5.47 ± 1.08 (5.40–5.53) 4.88 ± 1.69 (4.74–5.03) 4.46 ± 2.10 (3.86–5.06) 5.25 ± 1.37 (5.04–5.47)	<0.0001*	4.8 ± 2.1 (4.7–4.9) 5.0 ± 2.3 (4.7–5.2) 4.5 ± 2.0 (4.2–4.8) 4.9 ± 2.0 (4.8–5.0) 4.6 ± 2.1 (4.4–4.8) 4.0 ± 2.0 (3.5–4.6) 4.7 ± 2.2 (4.3–5.0)	0.004	4.9 ± 1.6 (4.8–4.9) 4.8 ± 1.8 (4.6–4.9) 4.8 ± 1.6 (4.6–5.1) 4.8 ± 1.5 (4.7–4.9) 5.06 ± 1.8 (4.9–5.2) 4.8 ± 2.1 (4.1–5.4) 4.8 ± 1.6 (4.6–5.1)		0.216
Medical education background (Total) Yes No	3.78 ± 2.2 (3.7–3.9) 4.4 ± 1.7 (4.2–4.7) 3.3 ± 2.4 (3.1–3.4)	<0.0001*	5.22 ± 1.41 (5.16–5.27) 5.60 ± 0.97 (5.54–5.66) 4.91 ± 1.61 (4.82–5.00)	<0.0001*	4.8 ± 2.1 (4.7–4.9) 4.8 ± 2.1 (4.7–4.9) 4.7 ± 2.1 (4.6–4.8)	0.318	4.9 ± 1.6 (4.8–4.9) 4.8 ± 1.4 (4.8–4.9) 4.9 ± 1.8 (4.8–5.0)		0.832
Household income(Total) Low (Under 50.000 SP*) Moderate 50.000–100.000 SP) Good (100.000–300.000 SP) High (Above 300.000 SP)	3.78 ± 2.2 (3.7–3.87) 3.87 ± 2.5 (3.57–4.2) 3.75 ± 2.4 (3.6–3.9) 4.02 ± 2 (3.6–3.8) 3.78 ± 2 (3.7–4.3)	0.463	5.22 ± 1.41 (5.16–5.27) 4.97 ± 1.61 (4.77–5.16) 5.12 ± 1.51 (5.03–5.22) 5.33 ± 1.28 (5.25–5.40) 5.48 ± 1.13 (5.30–5.67)	<0.0001*	4.8 ± 2.1 (4.7–4.9) 4.7 ± 2.2 (4.4–5.0) 4.8 ± 2.1 (4.6–4.9) 4.8 ± 2.0 (4.7–4.9) 4.5 ± 2.2 (4.1–4.8)	0.262	4.9 ± 1.6 (4.8–4.9) 5.1 ± 1.8 (4.9–5.4) 5.02 ± 1.7 (4.9–5.1) 4.7 ± 1.5 (4.6–4.8) 4.8 ± 1.4 (4.5–5.04)		<0.0001*
Chronic disease (Total) No Yes	3.78 ± 2.2 (3.7–3.8) 3.74 ± 2.1 (3.6–3.8) 4.1 ± 2.4 (3.8–4.4)	<0.0001*	5.22 ± 1.41 (5.16–5.27) 5.20 ± 1.56 (5.01–5.40) 5.22 ± 1.39 (5.16–5.28)	0.880	4.8 ± 2.1 (4.7–4.9) 4.8 ± 2.1 (4.7–4.9) 4.5 ± 2.4 (4.2–4.8)	0.015	4.9 ± 1.6 (4.8–4.9) 4.9 ± 1.6 (4.8–4.9) 4.8 ± 1.8 (4.6–5.1)		0.830

*p < 0.05.

Of the six predictor variables, only two were statistically significant: education level and the presence of medical education or practice (as shown in Table 4). University stage or above had 1.827 times higher odds to exhibit good knowledge toward COVID19 than primary or below the level of education. In addition, those with a medical practice or education were 2.83 times more likely than others to demonstrate accurate knowledge of COVID19.

**Table 4:** Binary logistic regression between the scales that assess knowledge toward COVID‐19 and antibiotics resistance, and demographic characteristics

	Knowledge of COVID‐19				Knowledge of antibiotics resistance			
Variable	OR	95% CI for B		*p*‐value	OR	95% CI for B		*p*‐value
		Lower	Upper			Lower	Upper	
Age (years) 18–29 (Ref) 30–49 Above 50	0.86 0.979	0.65 0.63	1.13 1.52	0.29 0.924	1.13 1.28	0.83 0.81	1.54 2.03	0.42 0.29
Gender (Male: Ref)	0.99	0.79	1.24	0.97	0.79	0.63	0.98	0.03*
Education Primary or below(Ref) Secondary Tertiary	1.76 1.82	1.236 1.296	2.51 2.57	0.002* 0.001*	0.70 0.68	0.48 0.47	1.04 0.99	0.076 0.044*
The job Full‐time (government) (Ref) Full‐time (private) Student Unemployed Retiree New graduated	0.67 1.12 0.88 0.69 0.91	0.44 0.77 0.62 0.35 0.55	1.0 1.61 1.23 1.36 1.49	0.052 0.53 0.45 0.28 0.71	0.82 0.82 1.14 1.3 0.96	0.52 0.56 0.79 0.64 0.58	1.29 1.20 1.64 2.6 1.6	0.397 0.314 0.474 0.458 0.897
Medical education Background (No: Ref)	2.83	2.182	3.66	<0.001*	2.05	1.59	2.64	<0.001*
Household income Low (Under 50.000 SP*) (Ref) Moderate (50.000–100.000 SP) Good (100.000–300.000 SP) High(Above 300.000 SP)	1.19 1.25 1.69	0.87 0.90 0.96	1.63 1.74 2.98	0.27 0.16 0.66	0.93 0.63 0.71	0.66 0.44 0.42	1.3 0.89 1.18	0.655 0.009* 0.187
Chronic disease (No: Ref)	1.35	0.95	1.91	0.089	1.37	0.98	1.9	0.065

*p < 0.05.

### 3.2 Knowledge of antibiotics use and resistance (*n* = 2406)

Table 5 The respondents' mean score is 3.77 (SD = 2.19, range = 0–10), as the overall proportion of correct answers is 37.7%. The vast majority of respondents could not answer more than six correctly out of 10, indicating poor knowledge of antibiotics resistance (59.6%). When asked whether using antibiotics would hasten healing from all illnesses, just 16.6% said it wouldn't. Remarkably, just 41% of participants realized that using antibiotics wouldn't prevent all illnesses, but 65.3% correctly identified how poor use of medicines would increase the development of antibiotic resistance (Table 2). One‐way ANOVA revealed differences in antibiotic resistance knowledge scores across genders, age groups, educational achievement, employment, medical training, family income, and chronic health conditions using one‐way ANOVA (Table 3).

**Table 5:** Descriptive data of knowledge of antibiotics use and resistance.

Item	Correct	Incorrect	Unsure
1. Over time, some bacteria strains may quickly change.	874 (35.2%)	585 (24.3%)	974 (40.5%)
2. The process of creating new vaccines and antibiotics is quick and easy.	338 (14.0%)	1285 (53.4%)	782 (32.5%)
3. Antibiotics can stop any illness.	590 (24.5%)	987 (41%)	829 (34.5%)
4. Antibiotics may hasten the healing process for any illness.	1189 (49.4%)	401 (16.6%)	816 (33.9%)
5. It is possible to modify the dosage of antibiotics without consulting a qualified healthcare provider.	396 (16.5%)	1452 (60.3%)	558 (23.2%)
6. Only bacterial infections can be treated with antibiotics.	939 (39.0%)	568 (23.6%)	899 (37.4%)
7. Death may result from antibiotic resistance.	1014 (42.1%)	293 (12.2%)	1099 (45.7%)
8. A resistant bacterial strain has the potential to generate pandemic occurrences comparable to COVID‐19.	1231 (51.2%)	138 (5.7%)	1037 (43.1)
9. Antibiotic resistance will spread faster as a result of improper antibiotic usage.	1572 (65.3%)	178 (7.4%)	656 (27.3%)
10. To avoid antibiotic resistance, good hand cleanliness is crucial.	1022 (42.5%)	637 (26.5%)	747 (31.0%)

The Knowledge of Antibiotic Usage test revealed a statistically significant difference between men and women,, in which males had a higher knowledge of 3.93 ± 2.3 (3.7‐4.1) more than females of 3.7 ± 2.2 (3.6‐3.8) (*p* = 0.023). Moreover, the individuals with a medical education background have a higher knowledge of antibiotics usage and resistance 4.4 ± 1.7 (4.2–4.7) compared to individuals without a medical education background of 3.3 ± 2.4 (3.1–3.4) (*p* < 0.0001). Moreover, people with chronic diseases have demonstrated a statistically significant higher knowledge of antibiotic and antibiotic resistance compared to people who were not diagnosed with any chronic disease (Table 3).

Furthermore, of the six predictor variables, four variables were statistically significant: gender, educational level, household income and medical background (as shown in Table 4). Females had 0.79 times lower odds of exhibiting good Knowledge of antibiotics resistance than Males. Individuals with a medical background or practice are 2.05 times more likely to be knowledgeable about antibiotic resistance than nonmedical groups.

### 3.3 Practice of preventive measures

The average practice grade was 4.82 (SD = 2.149, rang: 0–9), scoring an overall proportion of good practice reaching only 48.2%. Only 29.7% of respondents have always maintained a physical distance of at least 1 m from others, only 33.7% committed to washing hands for at least 20 s, and only 39.1% used facemasks in public areas. In contrast, most respondents (78.5%) were committed to closing mouth and nose when sneezing or coughing. This was the most applied preventive practice of all (Table 6).

**Table 6:** Detailed information on the use of preventative measures during the 2019 coronavirus disease (COVID‐19) pandemic

Item	Yes	No
1. Washing your hands often after touching things that you use a lot.	1686 (71.2%)	720 (28.8%)
2. Whenever you contact your eyes, nose, or mouth, wash your hands first.	1200 (50.7%)	1206 (49.3%)
3. Wash hand for at least 20 s	799 (33.7%)	1607 (66.3%)
4. Wear face mask in public area	929 (39.1%)	1477 (60.9%)
5. While sneezing or coughing, cover your mouth and nose.	1865 (78.5%)	929 (21.5%)
6. Always bring along sanitizer or wet wipes	1144 (48.1%)	1262 (51.9%)
7. Maintain a physical distance of at least 1 meter from people.	685 (29.7%)	1721 (70.3%)
8. Avoid crowded and narrow places	1519 (65.9%)	887 (34.1%)
9. Avoid chatting and speaking at close distance	973 (42.2%)	1433 (57.8%)
10. No handshakes and greetings with a hand on the chest are the only forms of physical contact allowed.	1361 (59%)	1045 (41%)

We used a one‐way ANOVA factor to compare the scores of different preventive practices against COVID19 across sexes, age category, educational attainment, employment, training in medicine, and family income, and chronic conditions (Table 3). Otherwise, a statistically significant difference was found between the age groups and practicing the preventive measures against COVID19, as shown in Table 3, in which the 30–49 age group has the highest score of 4.9 ± 2.05 (4.7–5.1) (*p* < 0.0001).

### 3.4 Attitudes about the new norm during the COVID‐19 epidemic

The majority of respondents agree that body temperature monitoring should be used in all public settings, and that having hand sanitizer readily available will promote regular hand washing. Most of the responses encouraged the mandatory wearing of facemasks in all public areas. Most participants admitted the importance of governmental and educational programs having a major role in facing pandemics (Table 7).

**Table 7:** Descriptive data of attitude toward new norm during the COVID‐19 pandemic

Item	Strongly disagree	Disagree	Neutral	Agree	Strongly agree
1. In all public places, body temperature monitoring should be used.	102 (4.2%)	340 (14.1%)	728 (30.2%)	747 (31%)	489 (20.3%)
2. The presence of hand sanitizer in public spaces will promote regular hand washing	35 (1.5%)	47 (2.0%)	181 (7.5%)	979 (40.7%)	1164 (48.4%)
3. All public spaces should require the use of face masks.	41 (1.7%)	145 (6.0%)	386 (16.0%)	815 (33.9%)	1019 (42.4%)
4. Home‐based work is beneficial and ought to be promoted.	104 (4.3%)	378 (15.7%)	664 (27.6%)	784 (32.6%)	476 (19.8%)
5. Distancing between tables in restaurants needs to continue	25 (1.0%)	77 (3.2%)	308 (12.8%)	1191 (49.5%)	805 (33.5)
6. All foreign arrivals should be subject to obligatory quarantine.	95 (3.9%)	342 (14.2%)	752 (31.2%)	749 (31.1%)	468 (19.5%)
7. Government‐sponsored ongoing education has better prepared me to deal with this epidemic.	25 (1.0%)	28 (1.2%)	173 (7.2%)	966 (40.1%)	1214 (50.5%)

### 3.5 The correlation between the fourth scale

We identified a statistically significant, moderate positive correlation between knowledge of COVID19 scores and knowledge of antibiotics scores, practice scores and attitude scores (*r* = 0.41, *p* < 0.001), (*r* = 0.042, *p* = 0.039) and (*r* = 0.23, *p* < 0.001), respectively (Table 8). We discovered a statistically significant, but weakly positive connection between antibiotic knowledge and attitude ratings (*r* = 0.25, *p* < 0.001) (Table 8). Furthermore, we found no statistically significant association, positive correlation between knowledge of antibiotics scores and practice scores (r = 0.16, *p* = 0.43), Table 7. However, we detected a statistically significant, weak positive correlation between practice scores and attitude scores (*r* = 0.51, *p* = 0.012) (Table 8).

**Table 8:** Correlation matrix (Spearman) of knowledge of COVID‐19, knowledge of antibiotics, attitude, practice scores

Correlations	Knowledge of COVID‐19 scores	Knowledge of antibiotics scores	Practice scores	Attitude scores
Knowledge of COVID‐19 scores	1	‐	‐	‐
Knowledge of antibiotics scores	0.41 (*p* < 0.001*)	1	‐	‐
Practice scores	0.042 (*p*‐value: 0.039*)	0.16 (*p*‐value: 0.43)	1	‐
Attitude scores	0.23 (*p* < 0.001*)	0.25 (*p* < 0.001*)	0.51 (*p*‐value: 0.012*)	1

*p < 0.05.

## 4 DISCUSSION

Numerous KAP investigations on COVID‐19 were carried out globally in an attempt to measure the efficacy of public health education systems. It is important to continuously observe the progressive COVID‐19 situation to address the actual knowledge gap in the public and to develop more effective educational methods. In our study, we found that the general population of Syria has a relatively good knowledge of COVID‐19 and moderate acceptance of the new norm. There was little awareness of antibiotic usage, resistance, and prophylactic procedures.

Like the vast majority of KAP studies in many countries,^23^, ^24^, ^26^, ^27^ However, about 40% of respondents were uncertain or mistakenly believed that COVID‐19 transmission only occurs through symptomatic persons as it is commonly known that COVID‐19 is constantly spreading through asymptomatic carriers, and,^28^ probable that some knowledge barrier prevented adequate understanding of transmission in public. Similarly, a Malaysian KAP study resulted in similar findings. Regarding the transmission of COVID‐19 without symptoms, Chang et al. have noted a notable degree of ambiguity.^23^ The proportion transmissibility of asymptomatic instances may be much lower than that of symptomatic ones, according to certain research, however^29^ the general people should constantly be made aware of such transmissibility. In contrast to other studies^23^, ^26^, ^27^ the youngest respondents among the Syrian population had a better mean score regarding knowledge of COVID‐19 than older groups. This suggests that the Syrian youth has slightly better accessibility to COVID‐19 information. Internet accessibility might be an impacting factor. Surprisingly, participants with secondary education had better COVID‐19 knowledge mean score than respondents with tertiary education. Thus, further studies may be required to investigate such findings. In congruence with the same studies,^23^, ^26^, ^27^ better household income is associated with better COVID‐19 knowledge.

Regarding knowledge of antibiotics usage and related resistance, the Syrian population had a significantly poor overall knowledge, with an overall average of 3.77 out of 10. We have observed a chaotic behavior from Syrians in terms of antibiotics usage. We also noted that antibiotics are randomly used as a result of self‐prescription incident or prescriptions from unqualified or unlicensed personnel. It is safe to presume that antibiotics usage in Syria is still subjected to cultural misbeliefs. We noticed a high level of uncertainty in respondents' answers. At this point, it is clear that the Syrian population needs more extensive education regardless of age, educational background, and household income. We realized that respondents with a medical background also scored poorly, with a total score of 4.4 out of 10, which is just a marginal improvement compared to respondents with no medical experience or practice, whose mean score was 3.3. Similar studies have shown unfavorable results and a low overall mean score in the use of antibiotics.^30^, ^31^ It may be interesting to investigate on why higher educational level and income are associated with lower antibiotics resistance knowledge. This may potentially reveal huge systematic blunder in the education system especially in health section. High‐income personals maybe depending on low creditable sources of information regarding antibiotics. With only 42.1% of respondents believing that antibiotic resistance could be fatal, more efforts are required to educate the public about the possible adverse effects of antibiotic resistance. Overall, Syrians were more aware of COVID‐19 than they were of antibiotic usage.

On the one hand, information about COVID‐19 has been spread to the public through all kinds of media daily since the beginning of the pandemic. Also, several rules and penalties have been enforced by governmental bodies. On the other hand, only a limited number of campaigns have talked about antibiotic resistance, and there were no legal penalties in this regard; thus, many penalties should be performed on the persons or pharmacists if they prescribe the antibiotics randomly. Furthermore, witnessing death and co‐morbidities of COVID‐19 may have affected the public interest in the problem. Nevertheless, more epidemiological studies are needed to determine the knowledge gap in these two health issues.

During the COVID‐19 epidemic, the Syrian population has shown a modest degree of receptivity toward new norms. Attitude overall average was 4.9 out of 7. Only 19.8% of respondents strongly agreed to pursue working from home as it is equally productive. This was understandable as very few Syrians have worked from home throughout the pandemic in the first place. Workers who worked from home, in contrast to common assumption, reported a reduction in overall physical and mental health status as well as an increase in the frequency of new physical and mental health conditions.^32^ Similar to parallel studies,^23^, ^24^ the vast majority agreed to the mandatory wearing of facemasks and body temperature measuring in public places.

As for the practice of preventive measures during the pandemic, the respondents scored poorly, with 4.8 out of 10. Half of the respondents do not wash hands before touching their eyes, nose, and mouth, about 60% will not wear masks in public. Wide educational campaigns have been held since the beginning of COVID‐19 outbreak. Despite that most people acknowledge the importance of wearing masks, we hypothesize that Syrians are facing more major issues like poverty, unemployment, and war, which all made the process of mask wearing insignificant. Also, no actual embedding of penalty system may have exaggerated this behavior. In addition, about 70% will not maintain physical distancing despite the desperate health calls throughout the pandemic. In a national Australian survey,^33^ Thomas et al. found that 50% of respondents who were not complying with physical distancing believed it was “unnecessary.” In another study in the Philippines, similar to our findings, Lau et al. noted that 70.8% of participants do not wear face masks.^34^ In conclusion, we can notice that there is a low level of compliance when practicing preventive measures in different ethnicities despite the difference in demography and overall socioeconomic status.

### 4.1 Limitations

Initially, despite its cost‐effectiveness and practicality, the cross‐sectional study design cannot prove causation. Moreover, through using uniform sample and achieving a rate of response of 99%, which is more than the usual response rate for organization questionnaire survey, this study's generalizability was enhanced. Because surveys were anonymous, there was no way to contact participants after they had finished their questionnaires to verify any unconventional answers. In addition, it is crucial to confirm the lack of generalizability of our study findings toward those in the older age group, with lower educational background, and those without internet access who will be left out in this study.

With these limitations, several steps were taken to increase the study's dependability. To increase the internal validity of study results, for instance, use a validated instrument in addition to controlling for confounding factors in the final version and sample from a wide range of research places. A preconceived sample size calculations are also performed to make sure that the project is effective.

## 5 CONCLUSION

Our study concluded that the Syrian population demonstrated good knowledge of COVID‐19 and moderate acceptance of the new norm. Knowledge regarding antibiotic use and resistance and practice of preventive measures was poor, which can encourage the health authorities to develop community education programs to increase public awareness of the usage of antibiotics and safety precautions during the COVID‐19 epidemic.

## AUTHOR CONTRIBUTIONS

**Sarya Swed**: Conceptualization; formal analysis; methodology; resources. **Sheikh Shoib**, **Mohammad B. Almoshantaf**, **Walaa Hasan**, **Yomna E. Dean**, **Yousef Tanas**, **Haidara Bohsas**, **Hidar Alibrahim**, **Mohammad M. Hasan**, **Weaam Ezzdean**, **Hazem S. Ghaith**, **Lina T. Khairy**, **Agyad Bakkour**, **Ali Hadi Hussein Muwaili**, **Fatima A. A. Abdelmajid**, **Mhd K. Albuni**, **Elias Battikh**, **Dhuha Hadi Hussein Muwaili**, **Rima Qattea**, **Karam R. Motawea**, **Bisher Sawaf**, **Nashaat Kamal Hamdy Elkalagi**, **Safaa M. A. Ahmed, Hani Aias**: Writing – review and editing.

## CONFLICT OF INTEREST

The authors declare no conflict of interest.

## ETHICS STATEMENT

All experimental procedures have been approved by institutional review boards and/or ethical licensing committees in Aleppo and Damascus.

## TRANSPARENCY STATEMENT

The lead author Mohammad Badr Almoshantaf affirms that this manuscript is an honest, accurate, and transparent account of the study being reported; that no important aspects of the study have been omitted; and that any discrepancies from the study as planned (and, if relevant, registered) have been explained.

## Supporting information

Supplementary information.

## Data Availability

The authors have accessible and saved all the information needed to draw the conclusion in this study. Any reasonable request will result in access to all data from the corresponding author.
