# Supplementary material for: Knowledge, attitudes, and practices related to COVID‐19 infection, related behavior, antibiotics usage, and resistance among Syrian population: A cross‐sectional study
Source: Health Sci Rep. 2022 Oct 3;5(6):e833. doi: 10.1002/hsr2.833 (PMC9528956; doi:10.1002/hsr2.833)
Supplement: Supplementary file 1 — Supplementary information. [file HSR2-5-0-s001.docx]

| Age (years) | 18-29  30-49  Above 50 |
| --- | --- |
| Gender | Male  Female |
| Education | Primary or below  Secondary  Tertiary |
| Occupation | Full time (government)  Full time (private)  Student  Unemployed  Retiree  New graduated |
| Medical education background | Yes  No |
|  | Household income  Bad (Under 50.000 SP*)  Moderate (50.000-100.000 SP)  Good (100.000-300.000 SP)  High(Above 300.000 SP) |
| Chronic disease | Yes  No |

***Demographic characteristics***

| **True** | **False** | **Unsure** | **Statements** | **NO.** |
| --- | --- | --- | --- | --- |
|  |  |  | The COVID-19 pandemic is virus origin | ● |
|  |  |  | The main clinical symptoms of COVID-19 are fever, cough, sore throat and difficulty breathing | ● |
|  |  |  | COVID-19 can affect anyone at any stage of life | ● |
|  |  |  | Elderly, child, people with co-morbidities and immunocompromised personnel are more susceptible to COVID-19 and develops more complications if infected | ● |
|  |  |  | COVID-19 virus is spread mainly through respiratory droplets | ● |
|  |  |  | Transmission of COVID-19 virus can only happen when a person developed symptoms | ● |
|  |  |  | COVID-19 virus strains can mutate over time | ● |

**Background Knowledge about COVID-19 Pandemic**

**Hand Hygiene:**

| **Yes** | **No** | **Preventive Measures** | **No.** |
| --- | --- | --- | --- |
|  |  | Frequent hand washing after in contact with frequent touched surfaces | ● |
|  |  | Wash your hands before and after touching eyes, nose and mouth | ● |
|  |  | 6 steps hand washing that lasts for at least 20 s | ● |

**Personal Care:**

| **Yes** | **No** | **Preventive Measures** | **No.** |
| --- | --- | --- | --- |
|  |  | Face mask wearing at crowded area, public transport | ● |
|  |  | Cough and sneeze etiquette | ● |
|  |  | Bringing along hand sanitizers or wipes whenever going out | ● |

**Social Interaction:**

| **Yes** | **No** | **Preventive Measures** | **No.** |
| --- | --- | --- | --- |
|  |  | Physical distancing at least 1 m | ● |
|  |  | Avoid crowded places | ● |
|  |  | Avoid talking in close distance | ● |
|  |  | Limit physical contact: no handshake policy, salam letak tangan di dada | ● |

**Section C: Preventive Measures during COVID-19 pandemic**

| **Yes** | **No** | **Unsure** | **Statement** | **No.** |
| --- | --- | --- | --- | --- |
| **General knowledge** | | | | |
|  |  |  | Bacteria strains can mutate rapidly in a short period of time | ● |
|  |  |  | Developments of new antimicrobials/vaccinations is simple and does not take up much time | ● |
| **Antibiotics use** | | | | |
|  |  |  | Antibiotic use can prevent all infections from getting worst | ● |
|  |  |  | Antibiotic use can help to fasten the recovery process | ● |
|  |  |  | Antibiotic dosage adjustment can be done based on severity of disease without seeking professional medical advice | ● |
|  |  |  | Antibiotic is used for bacterial infection only | ● |
| **Antibiotics resistance** | | | | |
|  |  |  | Antibiotic resistance can cause death | ● |
|  |  |  | Like COVID-19, a new resistant bacteria strain can cause similar or worst pandemic events | ● |
|  |  |  | Misuse of antibiotics will accelerate the antibiotic resistance process | ● |
|  |  |  | Hand hygiene practice is essential to prevent antibiotic resistance | ● |

**Section D: Knowledge towards Antibiotic Use and Antibiotic Resistance**

| **Strongly Agree** | **Agree** | **Neutral** | **Disagree** | **Strongly Disagree** | **Question** | **No.** |
| --- | --- | --- | --- | --- | --- | --- |
|  |  |  |  |  | Temperature screening should be continued at public areas and crowded areas | ● |
|  |  |  |  |  | Preparing more hand sanitizers at public areas will encourage frequent hand sanitizing | ● |
|  |  |  |  |  | Face mask wearing should be made mandatory to those suffering from respiratory tract infections | ● |
|  |  |  |  |  | Working from home is productive and should be encouraged | ● |
|  |  |  |  |  | Table distancing should be continued at food outlets | ● |
|  |  |  |  |  | Home quarantine should be made compulsory to all international travelers | ● |
|  |  |  |  |  | Continuous education on infectious disease by the government to public is essential to prevent a new outbreak | ● |

**Section E: Adapting to a new norm Post COVID-19 pandemic**
